# Mandrakes

**Published:** 1903-04

**Authors:** J. W. M’Garvey

**Affiliations:** Lexington, Ky.


### Mandrakes.

BY J. W. M'GARVEY, LEXINGTON, KY.

It may be safely said that the mandrake is the most curious plant in the vegetable kingdom. On account of the singular form of its roots, it has been an object of superstition from the earliest period of history. It is defined in Webster's dictionary as "A low plant, having a fleshy root, often forked, and supposed to resemble a man." If the author of that definition had seen the one now lying before me, he would not have used the words, "supposed to resemble a man," but he would have said, "having a root in the form of a human being." I have three specimens which were brought to me for use in the College of the Bible by G. N. Shishamian in the summer of 1888, of which I have ever since intended to publish a description, but have neglected it hitherto. One of them, the finest specimen, is in the form of a man with a child clasped in his arms. Its head is irregularly formed, the face being a little flattened, but the eyes, nose and mouth are distinctly marked. The arms project from regularly formed shoulders, and, passing around the child, terminate in the hands with five separate fingers. The left hand has the shape of a squirrel's paw, but the right has a palm, four fingers in a line, and a thumb diverging from the fore-finger at an angle of about twenty degrees. The lower limbs and the feet are well formed, except that the toes do not show. It stands erect on its right foot, but the left leg is bent at the knee to a right angle, so that the lower part of this leg is held in a horizontal position. It is about ten inches high. The face of the child shows above the arms that hold it, and its features are more naturally formed than those of the man. No one fails, at the first glane at this curious object, to see all the points of this description; and the first feeling, after gazing at it, is one of incredulity. Nothing could convince us that it grew in the ground, but the well-known history of the plant, and the fact is obvious on close inspection that it is a root, and that no knife has ever touched it. It has the color of a peanut pod, and it has the same unevenness of surface, the result of shrinkage in drying. It is wood, and somewhat resembles a briar-root. The other two specimens are twins. They are in the form of two human figures of equal size, facing each other, and the arms of each are clasped around the other. As they grew in the ground, little fibrous rootlets were attached to them on every side, by means of which they drew nutriment from the soil. These are seen in the cut given in Webster's dictionary, but they have been rubbed away from my specimens. The cut in the dictionary represents a very imperfect specimen, with legs and a human body, but no arms or head.

I have not been able to find in my reading an account of a specimen approaching the perfection of the one first described above. I have had it photographed, and if any of the readers of this article wish to see an exact representation of it, they can obtain the photograph for twenty-five cents by writing to Mr. W. E. Johns, photographer, of this city, or to myself. It is not at all surprising that a plant whose roof has such a form should be an object of superstition. The plant above ground resembles the lettuce, and it bears a round fruit about the size of a large cherry, which is yellow when ripe, and which has a narcotic effect when eaten. Rachel believed that the use of it, whether by eating the fruit or by some use of the root, does not appear, would cure barrenness, and hence her anxiety to obtain those which were found by Leah's son, Reuben (Gen. xxx:14-25); but the superstition should have been corrected by her experience, for Leah, who parted from them, bore three children before Rachel, who obtained them, bore one. The writer in Webster's dictionary says that it was supposed to have animal life, and to cry out when pulled up, and he quotes from Shakespeare the couplet:

> "And shrieks like mandrakes torn out of the earth,
> That living mortals, hearing them, run mad."

Josephus gives an account of it under another name. After describing a valley, which he calls Baaras, which is none other than the deep narrow gorge entering the Dead Sea from the east, and containing the celebrated hot springs of Callirhoe, he says: "But still in that valley which encompasses the city on the north side, there is a certain place Baaras, which produces a root of the same name with itself. Its color it like flame, and towards evening it sends out a certain ray like lightning. It is not easily taken by those that would take it, but recedes from their hands, nor will it be taken quietly until either chamber lie or a woman's blood be poured upon it; nay, even then it is certain death to those who touch it, unless one take and hang the root itself down from his hand, and so carry it away. It may also be taken another way without danger, which is this: They dig a trench quite round about it, till the hidden part of the root be very small, they then tie a dog to it, and when the dog tries hard to follow him who tied him, the root is easily plucked up; but the dog dies immediately, as if it were in the place of the man who would take the plant away; nor after this need any one be afraid of taking it into his hands. Yet, after all this pains in getting it, it is only valuable on account of one virtue which it hath, that if it only be brought to sick persons, it quickly drives away those called demons, which are no other than the spirits of the wicked that enter into them that are alive, and kill them unless they can obtain some help against them." (War, book vii, chap. vi, sec. 3).

It is very clear from this account that Josephus had never seen one of the plants, or its roots, but that he tells the tale as it was told to him. Similar superstitions exist to this day in Turkey. The few roots which are found in Asia Minor and Syria are promptly bought up by the wives of rich men, and carefully kept among their treasures. When it was known in Constantinople that Brother Shishamian had the three which he kindly brought to me, his wife was besieged by various women of the place to let them have at least a small piece of one, as a guard against disease. He found these three in the hands of a poor man in the vicinity of Tarsus in Silicia, and prevailed on him to part with them. If there is another specimen in this country, I have not heard of it, and I doubt whether another equal to the best of these three has been found in modern times. Send for the photograph.

